# Supplementary material for: Tissue‐resident macrophages actively suppress IL‐1beta release via a reactive prostanoid/IL‐10 pathway
Source: EMBO J. 2020 Jun 2;39(14):e103454. doi: 10.15252/embj.2019103454 (PMC7360975; doi:10.15252/embj.2019103454)
Supplement: Supplementary file 1 — Expanded View Figures PDF [file EMBJ-39-e103454-s001.pdf]

Expanded View Figures

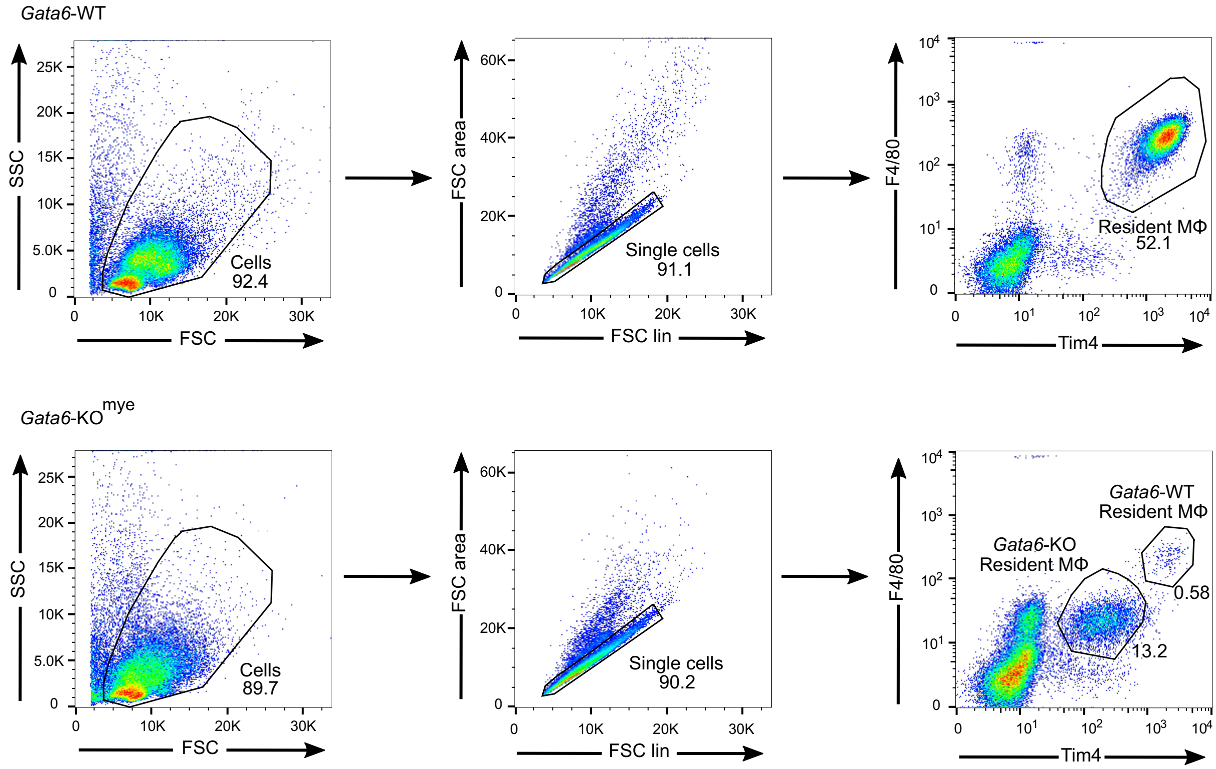

Figure EV1. Representative gating strategy of pMΦ from Gata6-WT and Gata6-KO^{mye} mice.

First, dead cells are excluded based on FSC and SSC value; then, single cells are gated based on FCS area and FCS linear value. pMΦ are then identified as F4/80^{hi}Tim4^{+} (Gata6-WT) or F4/80^{+}Tim4^{+} (Gata6-KO^{mye}).

© 2020 The Authors

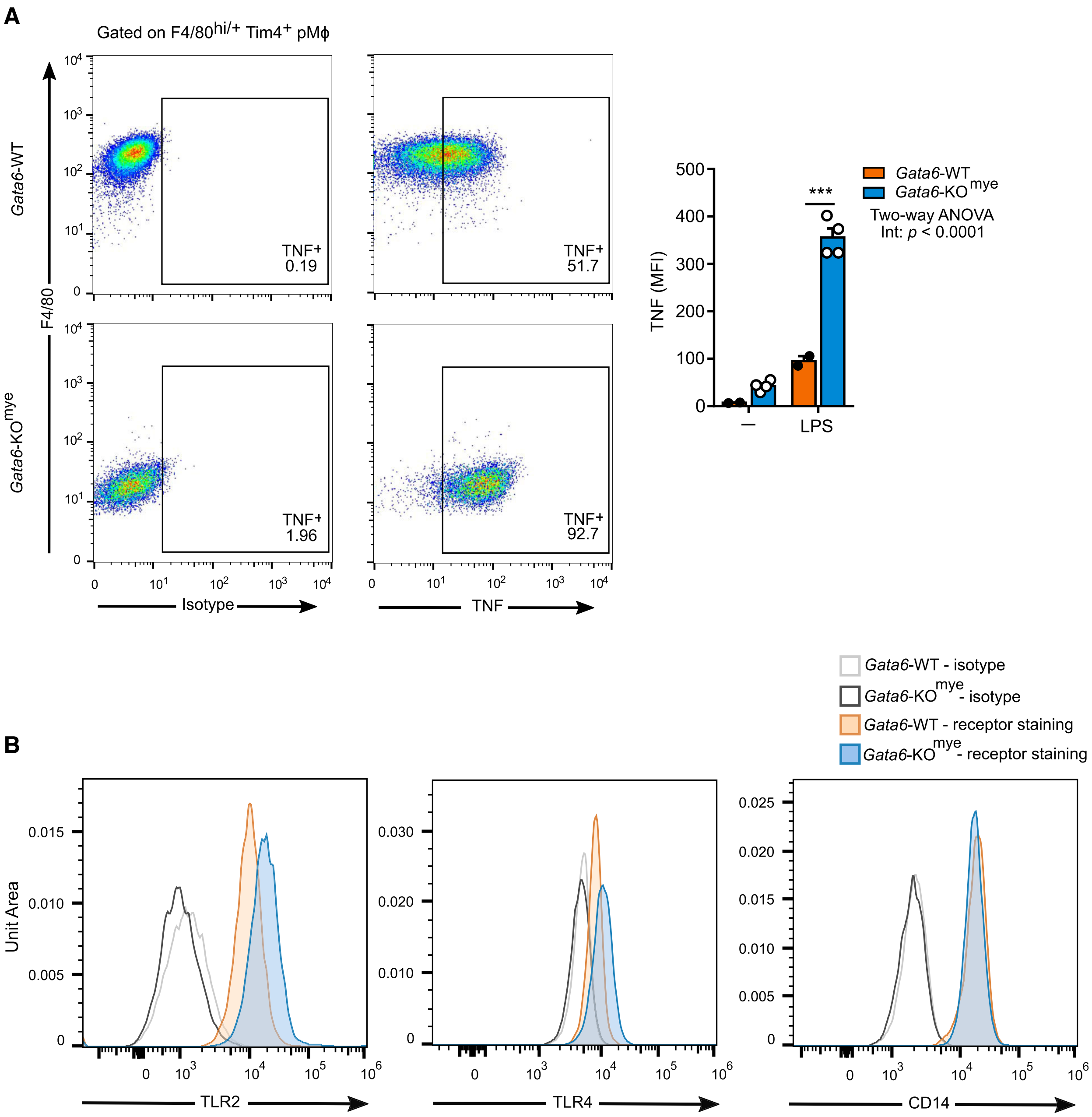

Figure EV2. Flow cytometry analysis of intracellular TNF and extracellular TLR2, TLR4 and CD14 expression.

A Representative flow cytometry analysis of TNF expression in Gata6-WT and Gata6-KO^{mye} pMΦ stimulated for 3 h with 100 ng/ml LPS and 0.1 % (v/v) GolgiBlock. Data shown are representative of at least three independent experiments. Data are expressed as mean ± SEM. Two-way ANOVA analysis followed by Tukey's multiple comparison post-test was performed. ***$P < 0.001$.

B Histogram overlays of flow cytometry analysis of cell surface expression of TLR2, TLR4 and CD14 of Gata6-WT and Gata6-KO^{mye} pMΦ.

© 2020 The Authors

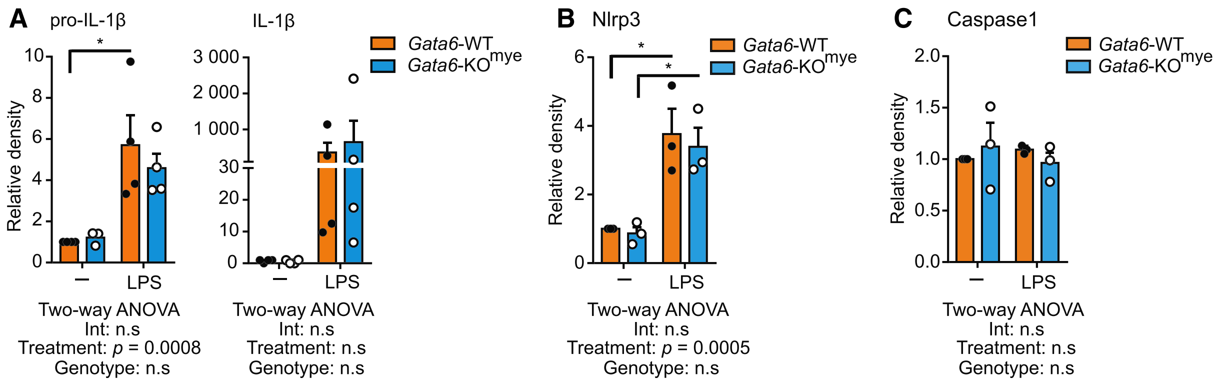

Figure EV3. Western blot quantification of the immature and mature IL-1β and the canonical inflammasome machinery.

Western blot quantification of pro-IL-1β and mature IL-1β (A), Nlrp3 (B) and caspase1 (C) of Gata6-WT and Gata6-KO^{mye} pMΦ, unstimulated or stimulated overnight with 100 ng/ml LPS. Results showed are pooled from three independent experiments, normalized to Gata6-WT unstimulated sample and are expressed as mean ± SEM. Two-way ANOVA analysis followed by Tukey's multiple comparison post-test was performed. *$P < 0.05$.

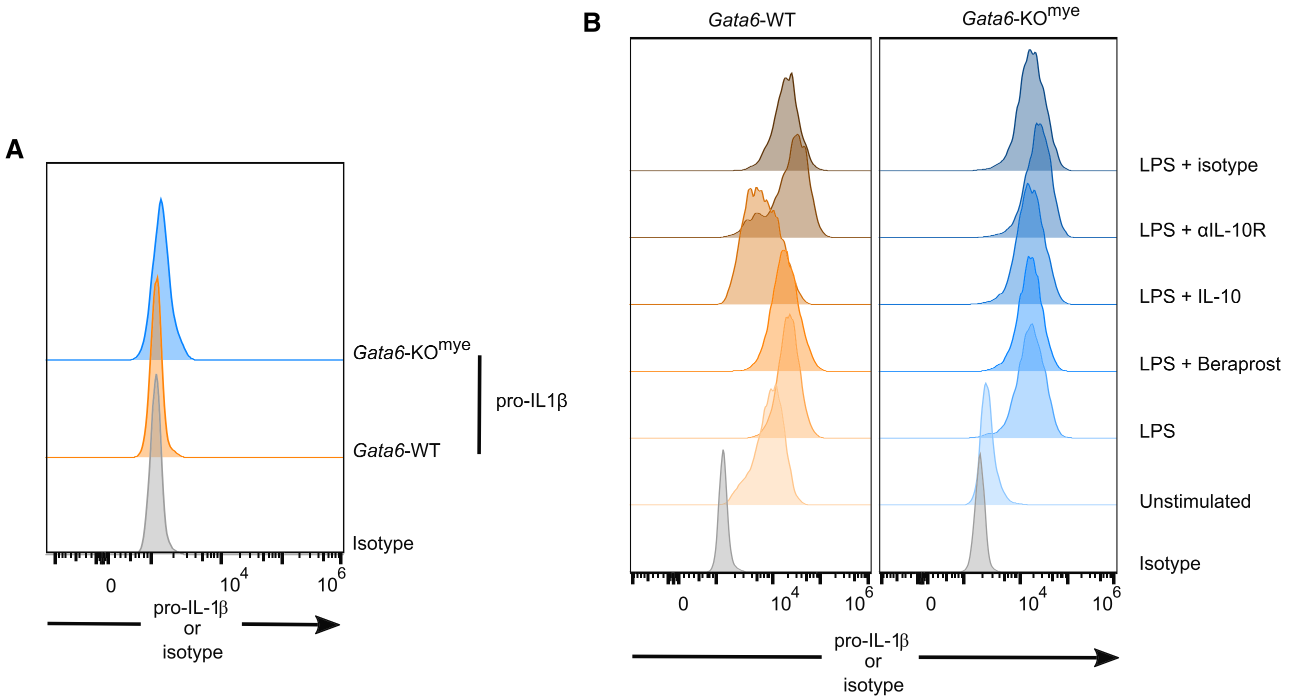

Figure EV4. Flow cytometry analysis of intracellular pro-IL-1β.

Flow cytometry analysis of intracellular pro-IL-1β content in Gata6-WT and Gata6-KO^{mye} pMΦ freshly isolated (A) or after 16 h incubation unstimulated or stimulated with 100 ng/ml ultrapure LPS, 10 µM beraprost, 10 ng/ml IL-10, 5 µg/ml αIL-10R or 5 µg/ml isotype (B). Data shown are representative of n = 6–13 individual mice.

 © 2020 The Authors

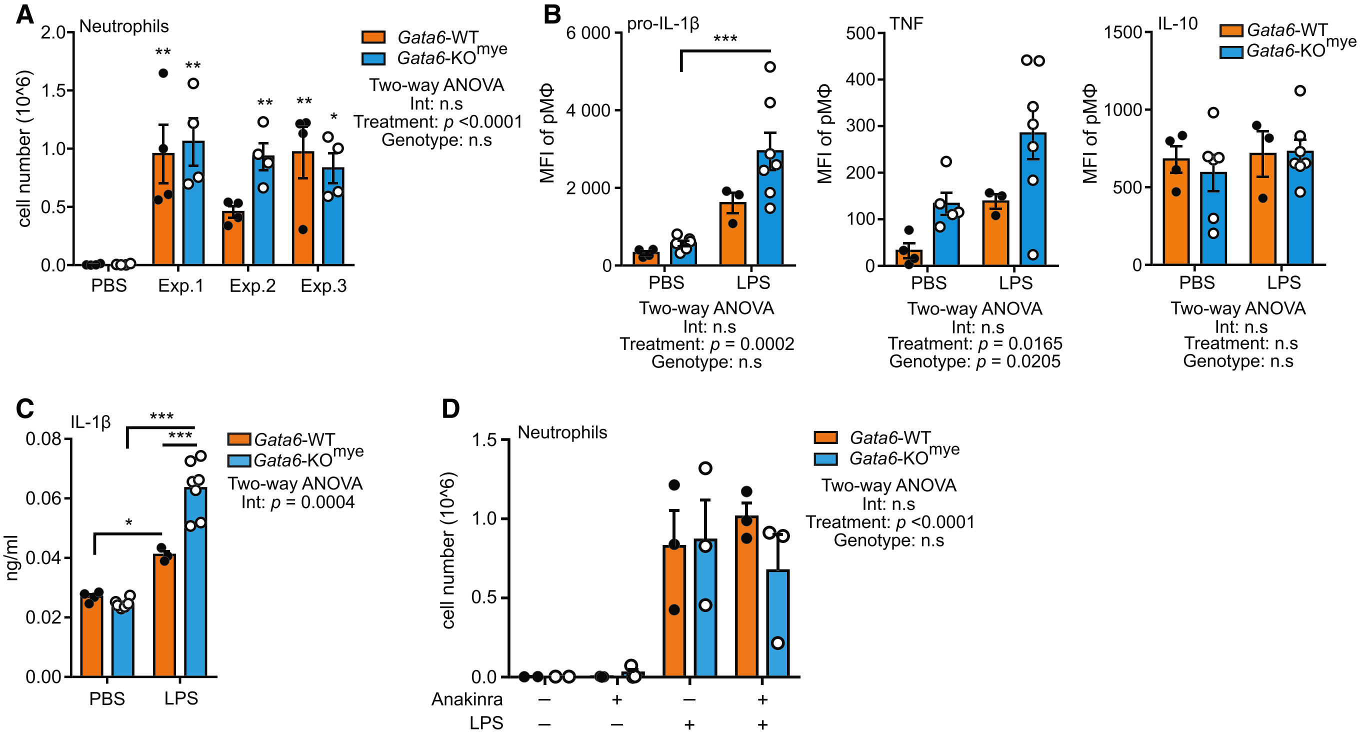

Figure EV5. Flow cytometry analysis of LPS-induced peritonitis in Gata6-WT and Gata6-KO^{mye} mice.

A Flow cytomerty analysis of neutrophil (Ly6G^{+} CD11b^{+}) number recruited to the peritoneal cavity of Gata6-WT and Gata6-KO^{mye} mice 4 h after i.p injection of 1 ng ultra-pure LPS. Three independent experiments are shown, n = 4 mice per group. Significance are indicated by * over the bar, compared to the corresponding control (Gata6-WT or Gata6-KO^{mye} PBS injected).

B Flow cytometry analysis of intracellular cytokines in Gata6-WT and Gata6-KO^{mye} pMΦ, 3 h after i.p injection of PBS or 1 ng ultra-pure LPS.

C ELISA analysis of IL-1β contained in the peritoneal fluid of Gata6-WT and Gata6-KO^{mye} mice, 3 h after i.p injection of PBS or 1 ng ultra-pure LPS. n = 3–6 mice per group.

D Flow cytomerty analysis of neutrophil (Ly6G^{+} CD11b^{+}) number recruited to the peritoneal cavity of Gata6-WT and Gata6-KO^{mye} mice 4 h after i.p injection of 1 ng ultra-pure LPS combined or not with 50 mg/ml Anakinra, n = 2–3 mice per group. Results are expressed as mean ± SEM. Two-way ANOVA analysis followed by Tukey's multiple comparison post-test was performed. *P < 0.05, **P < 0.01, ***P < 0.001.

© 2020 The Authors
